# Effects of botulinum toxin serotype A on sleep problems in children with cerebral palsy and on mothers’ sleep quality and depression

**DOI:** 10.17712/nsj.2016.4.20160207

**Published:** 2016-10

**Authors:** Vildan Binay Safer, Sibel Ozbudak Demir, Esma Ozkan, Fulya Demircioglu Guneri

**Affiliations:** *From Ankara Physical Medicine Rehabilitation Training and Research Hospital, Ankara, Turkey*

## Abstract

**Objective:**

To evaluate botulinum toxin serotype A (BoNT-A) effects on sleep problems in children with cerebral palsy (CP) and on mothers’ sleep quality and depression at multiple time points.

**Methods:**

This is a single center, cross sectional, and observational study was conducted to assess children with CP who were admitted. We recruited children with CP who were admitted to Ministry of Health Physical Medicine and Rehabilitation Training and Research Hospital, Ankara, Turkey between September 2012 and April 2014 for the BoNT-A injection for lower limb spasticity. Sleep quality of children with CP were determined at baseline and at the first, third and sixth month after the BoNT-A injection. Sleep quality Pittsburgh Sleep Quality Index (PSQI) and depression (by Beck Depression Inventory-II Turkish version) in mothers were also assessed.

**Results:**

Twenty-four children with CP (7.05±2.69 years) underwent final assessment. Their bedtime resistance (11.71±3.26 versus (vs) 10±2.75, *p*<0.01), sleep anxiety (8.00±2.57 vs. 7.13±2.27, *p*=0.046) and daytime sleepiness (11.67±2.14 vs. 10.25±1.96, *p*<0.01) were significantly improved in the first month after the BoNT-A injection. In accordance with this, PSQI and BDI scores of the mothers decreased in the first month after the BoNT-A injection. Thereafter, BDI scores continued to decrease, whereas PSQI slightly increased in the third month.

**Conclusions:**

The BoNT-A injection for spasticity in children with CP may have the potential to improve sleep quality in children with CP and their primary caregiver, the mother, as well as to reduce depression in the mother.

Cerebral palsy (CP) in childhood is a leading cause of lifelong disability with prevalence ranging from 1.7 to 2.0 per 1000 live births in the developing world.^1^ The primary caregiver of a child with CP, usually the mother, is at increased risk for impaired psychology due to the child’s behavior and the demands of caregiving.^2^ Sleep problems are 4 times more prevalent among children with CP compared with those with typical development.^3^ The sleep habit of the child is a key factor in caregiver burden.^2^ And also, the sleep quality of the caregiver significantly correlates with sleep disturbance of the child with CP.^4^ Moreover, sleep disturbance of the child with CP may be causatively associated with caregiver depression.^4^ An earlier study reported that 39.8% of children with CP have at least one sleep problem per night that requires caregiver attention, resulting in impaired daytime activity for the caregiver.^5^

Furthermore, spasticity, pain and epilepsy are associated with the increased prevalence of disturbed sleep in CP.^3^ Spasticity is the most common clinical features of hypertonia, accounting for up to 80% of children with CP.^6^ Botulinum toxin serotype A (BoNT-A) is a well-established, clinically effective, safe and licensed pharmacological agent for lower limb spasticity.^7^ The main effect of BoNT-A in spasticity results from interrupting neuromuscular conduction, which lasts for approximately 3 months.^8^ This treatment has been demonstrated to have the potential to improve gait pattern, positioning and ease in care, while reducing pain.^7^,^9^-^12^ Positive effects of the BoNT-A injection in children with CP have previously been reported regarding settling more easily to sleep, and reducing the frequency of night-time waking and the need for turning because of hip discomfort. However, these results were based on the comments of parents rather than specific study outcomes.^12^ BoNT-A effects on caregiver burden, specifically depression in the mother, have not been previously assessed. The purpose of this study is to prospectively investigate the effects of the BoNT-A injection on sleep problems in the child, and sleep quality and depression in the mother, at multiple time points.

## Methods

### Participants

The Medical Ethics Committee of the Ministry of Health Physical Medicine and Rehabilitation Training and Research Hospital, Ankara Turkey gave ethical approval for this study with institutional review board reference number of “B.10.4.İSM.4.06.23.34.904.02/3963” and the study was hold according to principles of Helsinki Declaration. Children with CP who were admitted to our center from September 2012 to April 2014 for the BoNT-A injection for lower limb spasticity were recruited and evaluated in this single centered, cross sectional, observational study.

Inclusion criteria were as follows: (1) CP equivalent to Modified Ashworth Scale levels II or III and (2) the mother is the primary caregiver. Children were excluded if (1) they had undergone medication change, surgery or the BoNT-A injection during the previous 6 months, (2) the mother was on antidepressant medication or psychotherapy or any other intervention for depression, (3) they had a sibling with disability or (4) no formal consent was given by caregivers.

All the participants and caregivers were evaluated through a standard assessment. The clinical history taken for the children comprised demographic data, the predominant type of motor impairment (spastic diplegia, spastic hemiplegia, spastic quadriplegia, dystonic/dyskinetic CP and mixed type), factors associated with the development of sleep problems (intellectual disability, epilepsy and visual impairment), medication history, dose of BoNT-A, use of night orthoses, a standardized physical examination, height (m), weight (kg), body mass index (kg/m^2^), severity of CP as measured by the Gross Motor Function Classification System (GMFCS) level and muscle spasticity according to the modified Ashworth scale (MAS). For the mothers, demographic data, years of education, occupation and medication history were recorded.

### Measurements

All measurements were performed following a standard procedure at baseline and then 1, 3 and 6 months after BoNT-A injections. The baseline assessments were made during face-to-face interviews, whereas the other assessments at 1, 3 and 6 months were conducted over the telephone. All measurements were conducted with the primary caregiver, the mother.

### Sleep quality of children

The Children’s Sleep Habits Questionnaire (CSHQ) abbreviated form, consists of 33 questions in 8 domains (parasomnia, bedtime resistance, sleep duration, sleep anxiety, sleep onset delay, sleep disordered breathing, night waking, and daytime sleepiness), and is used to evaluate the sleep quality of the children.^4^ Each scored item is rated on a 3-point scale as occurring “usually” (>5 times/week), “sometimes” (2–4 times/week), or “rarely” (<1 times/week). A Total sleep disturbances score is calculated as the sum of all CSHQ scored questions, and can range from 33 to 99.^4^ The CSHQ cut-off point as a predictor for children with a clinical sleep problem was 41. This cut-off point has a sensitivity of 0.80 and specificity of 0.72.^4^ The validity and reliability of the Turkish version of CSHQ was reported by Fis et al^13^ with construct validity indicated that the total sleep scores did not differ by age and gender (*p*>0.05) and also Cronbach’s alpha=.78.^13^ Higher scores indicate the presence of greater sleep problems.

### Sleep quality of mothers

The Turkish version of Pittsburgh Sleep Quality Index (PSQI) was used to determine sleep quality and disturbance of the mothers over the previous month. The PSQI Turkish version has 24 individual items, each item in the scale is scored between 0 and 3 (no difficulty to severe difficulty). Seven component scores (sleep latency, habitual sleep efficiency, sleep quality, sleep duration, daytime dysfunction, sleep disturbance and use of sleep medication) are evaluated in the first 19 self-answered questions.^14^ The validity and reliability of the Turkish version of the scale was reported with pearson correlation co-efficiency ranges between 0.93- 0.98 and Cronbach’s alpha=.80.^14^ A total score >5 indicates poor sleep quality.

### Depression in mothers

The Beck Depression Inventory-II Turkish version (BDI-II-TR) was used to evaluate the presence and severity of depressive symptoms in the mothers. The BDI-II Turkish version comprises 21 items, describing depression symptoms as specified in the Diagnostic and Statistical Manual of Mental Disorders IV. The reliability of BDI-II-TR (Cronbach’s alpha=.90) and its cut-off scores (0–12 for minimal depression, 13–18 for mild, 19–28 for moderate and 29–63 for severe) were reported by Kapci et al.^15^

### Statistical analysis

All data were anonymized and recorded on a password protected Microsoft Excel spreadsheet and analyzed using the Statistical Package for Social Sciences Version 20 (SPSS Inc., Chicago, IL, USA). Data were presented as percentage for percentage and count for categorical variables and mean±standard deviations for continuous data. The Kolmogorov–Smirnow test was used to determine the distribution characteristic of variables. Intragroup changes between two time points were analyzed using a paired Student t-test or Wilcoxon signed-rank test as appropriate. The baseline associations between CSHQ, PSQI and BDI-II-TR were investigated using the Pearson correlation coefficient. Differences were considered significant at *p*<0.05.

## Results

**Figure 1:**
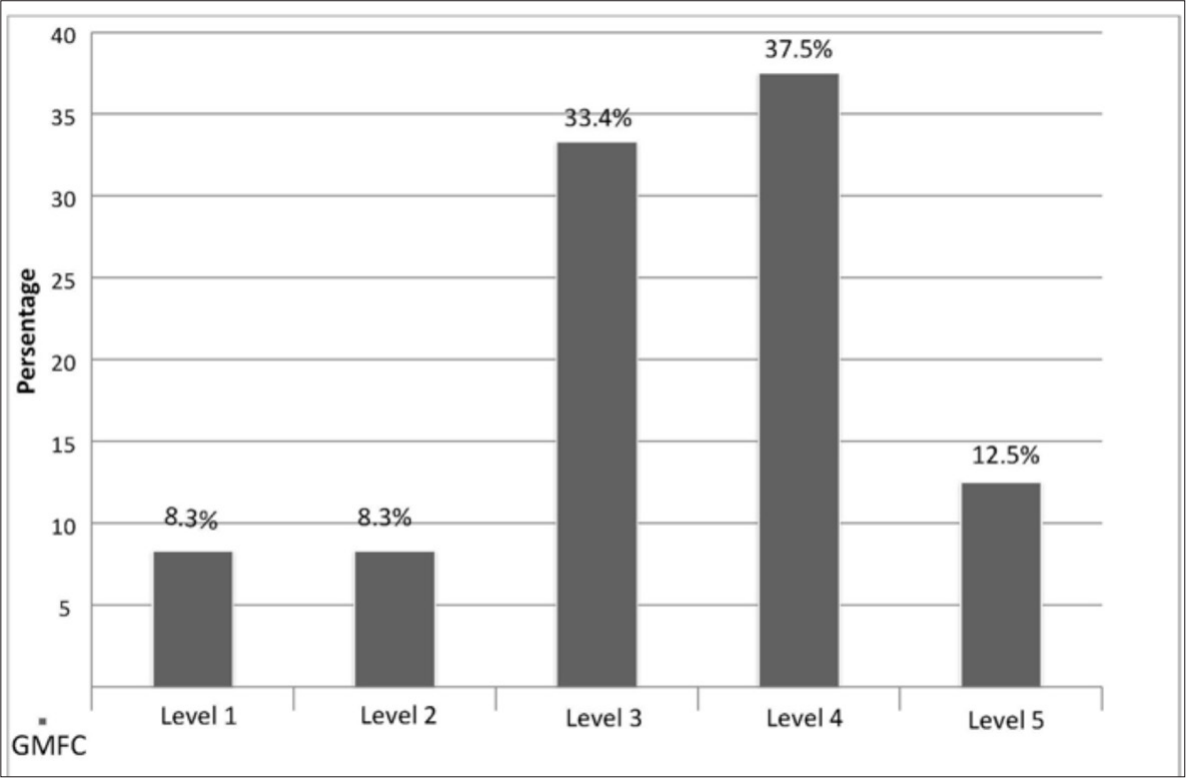
Gross motor function classification system levels of children with cerebral palsy at baseline.

**Figure 2:**
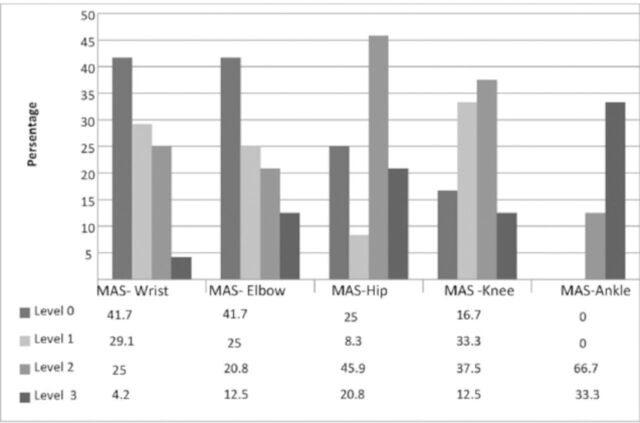
Modified Ashworh Scale levels of children with cerebral palsy at baseline. MAS - Modified Ashworh Scale

Over the 2-year period from September 2012 to April 2014, 30 children with CP (15 boys and 15 girls) and their primary caregivers who were their mothers recruited. Six children (3 boys and 3 girls) dropped out in the follow-up period, the final analyses were performed on 24 children and their mothers. The mean (SD) ages of the children and mothers were 7.05±2.69 (3.00–12.92) and 33.46±8.08 (21–47) years. Seventeen children had spastic diplegia, 4 had spastic hemiplegia and 3 had mixed type CP. Four children (16.6%) were at GMFCS level I and II, 8 (33.3%) were at GMFCS III, 9 (37.5%) were at GMFCS IV and 3 (12.55%) were at GMFCS V. The rate of use of night-time orthoses was 83.3% (n=20). Other demographic features of the children and mothers are summarized in **Table 1**; MAS and GMFC levels are shown in **Figures 1 and 2**.

**Table 1:** Characteristics of the children with cerebral palsy and mother.

Children characteristics	Baseline	Total CSHQ score
Age, mean±SD (years)	7.05±2.69	
Weight, mean±SD (kg)	23.51±9.95	
Sex Male/Female n (%)	13 (54.2) / 11 (45.8)	NS
*Gestation n (%)*		
Preterm	12 (50)	
37–42 weeks	12 (50)	NS
Hearing impairment	2 (8.3)	NS
Visual impairment	5 (20.9)	NS
Cognitive impairment	5 (20.9)	NS
Epilepsy	10 (41.7)	NS
Urinary incontinence	10 (41.7)	NS
Faecal incontinence	12 (50)	NS
Night time splint use	20 (83.3)	NS
Baclophen use	4 (16.7)	NS
BoNT-A dose for UL	1.63±3.59	
BoNT-A dose for LL	8.87±4.13	
Total BoNT-A dose	10.5±4.03	
*Mother characteristics mean±SD*		
Age, years	33.46±8.08	
Education, years	6.34±2.24	
Married	22 (91.7)	0.03*

CSHQ - Children’s Sleep Habits Questionnaire, BoNT-A - Botulinum toxin serotype A, UL - upper Limb, LL - lower limb, y - years, SD - standard deviations, n - numbers, NS - not significant,

* Mann–Whitney U test

### Sleep quality of children and mothers

**Figure 3:**
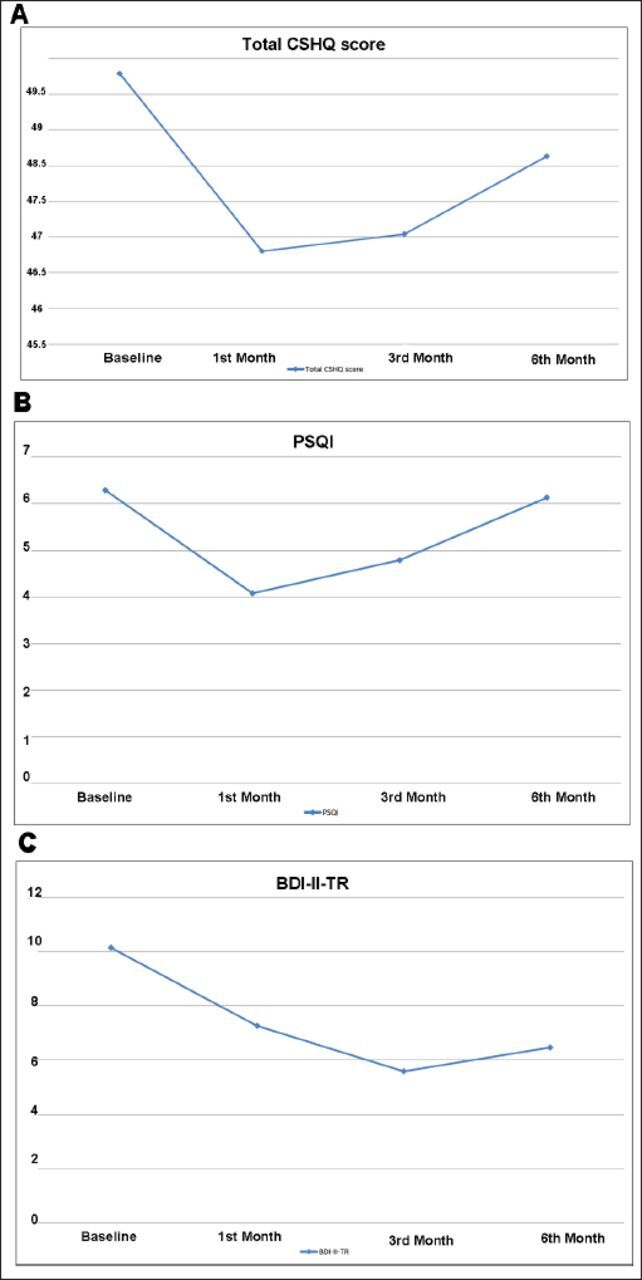
The scores of **a**) CSHQ of children with cerebral palsy at multiple time points, **b**) Pittsburgh Sleep Quality Index of mothers at multiple time points, **c**) Beck Depression Inventory-II Turkish of mothers at multiple time points. CSHQ - Children’s Sleep Habits Questionnaire, PSQI - Pittsburgh Sleep Quality Index, BDI-II-TR - Beck Depression Inventory-II Turkish

**Figure 4:**
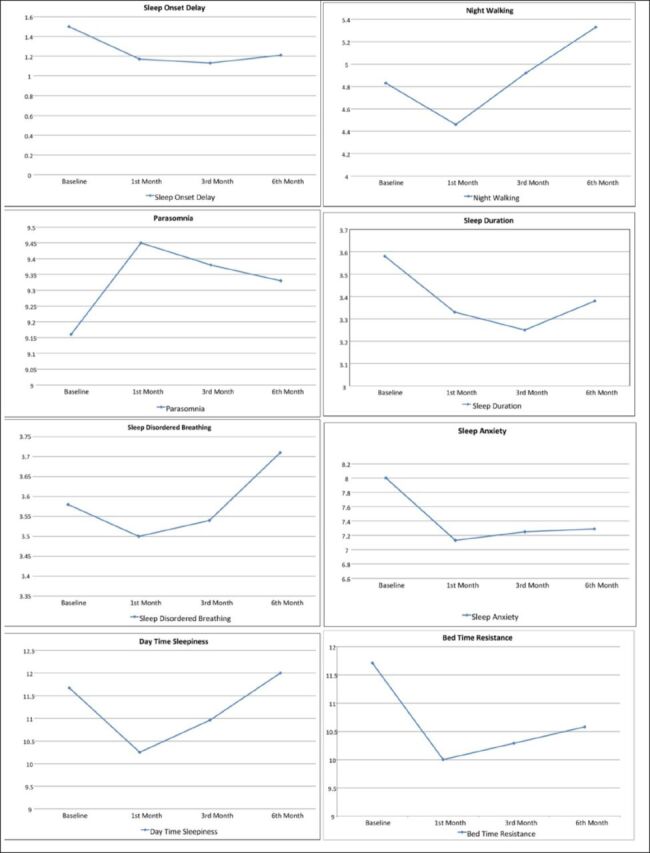
Total Children’s Sleep Habits Questionnaire subscale scores of children with cerebral palsy at multiple time points.

Children’s Sleep Habits Questionnaire findings are listed in **Table 2**. An abnormal total sleep score was observed in 79.2% (n=19) of the children with CP. Although a moderate correlation was observed at baseline between CSHQ scores of children with CP and PSQI of mothers (r=0.36), it was not significant (*p*=0.08). In addition, the total CSHQ score and all subscale scores except those for parasomnia decreased in the first month after the BoNT-A injection; however, only bedtime resistance (11.71±3.26 vs. 10±2.75; *p*<0.01), sleep anxiety (8.0±2.57 vs. 7.13±2.27; *p*=0.046) and daytime sleepiness scores (11.67±2.14 vs. 10.25±1.96; *p*<0.01) significantly improved. In accordance with this, PSQI scores of mothers (6.29±3.24 vs. 4.08± 2.34; *p*≤0.001) significantly decreased in the first month. Similarly, in the third month of treatment, the total CSHQ score and all subscale scores except for parasomnia were lower than the baseline scores but tended to have increased compared with the scores in the first month. Furthermore, compared with baseline, the change in PSQI scores was significant in the third month assessment. The result of the sixth-month evaluation demonstrated that PSQI, CSHQ and all CSHQ subscale scores except for parasomnia were increased compared with the third month assessment (**Figures 3 A-B and 4**). Although parasomnia scores were observed to be higher than baseline at all time points, none of these differences were significant.

**Table 2:** Changes over the time periods in the total CSHQ scores of children with cerebral palsy and PSQI and BDI-II-TR scores of mothers.

Variables	Baseline	First month	Third month	Sixth month	p1	p2	p3
	Mean±SD			
Total CSHQ score	49.29±6.45	46.8±6.2	47.04±5.782	48.13±5.551	NS	NS	NS
Bedtime resistance	11.71±3.26	10±2.75	10.29±2.69	10.58±2.45	0.009**	NS	NS
Sleep onset delay	1.5±0.72	1.17±0.48	1.13±0.34	1.21±0.41	NS	NS	NS
Sleep duration	3.58±1.32	3.33±0.56	3.25±0.53	3.38±0.77	NS	NS	NS
Sleep anxiety	8.0±2.57	7.13±2.27	7.25±2.54	7.29±2.33	0.046**	NS	NS
Night waking	4.83±1.05	4.46±1.28	4.92±1.28	5.33±1.4	NS	NS	NS
Parasomnia	9.16±2.16	9.45±1.79	9.38±1.79	9.33±1.97	NS	NS	NS
Sleep disordered breathing	3.58±0.93	3.5±0.72	3.54±0.88	3.71±0.91	NS	NS	NS
Day time sleepiness	11.67±2.14	10.25±1.96	10.96±1.83	12.0±2.65	0.007**	NS	NS
PSQI	6.29±3.24	4.08±2.34	4.79±2.65	6.13±3.33	<0.001**	0.028*	NS
BDI-II-TR	10.13±10.23	7.25±6.27	5.58±4.22	6.46±3.99	NS	0.014**	0.039**

CSHQ - Children’s Sleep Habits Questionnaire, PSQI - Pittsburgh Sleep Quality Index, BDI-II-TR - Beck Depression Inventory-II Turkish version, NS - not significant, p1 - baseline versus first month, p2 - baseline versus third month, p3 - baseline versus sixth month,

* paired Student t-test;

** related sample Wilcoxon signed-rank test

### Depression in mothers

The baseline BDI-II-TR scores of mothers revealed that 3 (12.5%) had moderate depression, 3 (12.5%) had severe depression and 18 (75%) had minimal depressive symptoms. At baseline, moderate significant correlations were found between the mothers’ BDI-II-TR and PSQI scores (r=0.57, *p*<0.001). In addition, the baseline BDI-II-TR scores of the mothers significantly correlated with the baseline CSHQ scores of children with CP (r=0.47, *p*<0.05). During follow-up, BDI-II-TR scores decreased for the first and third months and then slightly increased in the sixth month; nevertheless, BDI-II-TR scores at the third and sixth months were significantly lower than at baseline assessment (**Table 2, Figure 3C**).

## Discussion

This study provides information on the variation in level of depression of mothers at multiple time points after the BoNT-A injection. According to our results, BoNT-A injections for spasticity in children with CP have the potential to improve the sleep quality of mothers and children. In addition, the effects of BoNT-A were shown to be at their maximum in the first month following injection,^16^ which may be related to the improvement found in the depressive symptoms of mothers.

Earlier studies have shown that up to 35% of children with CP have sleep disorders.^17^,^18^ Several factors such as epilepsy,^17^,^18^ severe visual impairment^18^ and having a single parent^18^ have been shown to be related to sleep disorders in children with CP.^18^ The results of this study did not show relationship between epilepsy, severe visual impairment and sleep disorders. This might be due to our study population, which only consisted of only 1 child with severe visual impairment and 10 children with epilepsy, so relatively small sample size of these conditions might have failed to show a relationship between severe visual impairment, epilepsy and sleep disorders. However, all 11 of these children had sleep problems according to their CSHQ scores, which were above the cut-off value. Meanwhile, the baseline data assessment of the current work confirms that having a single parent is related to sleep problems in children with CP. This is well known that parents of children with development disabilities have an increased risk of divorce than parents of children without.^19^,^20^ Moreover, mothers who had children with cerebral palsy seems to have higher risk of marital disruption than mothers of healthy children.^21^ In this content, our observation was critical due to show potential negative effect of sleep problems of children with CP on risks of marital disruption.

Our baseline results underline the correlation between the sleep quality of children with CP and maternal depression. However, the observed correlation between the CSHQ score of the children and the PSQI score of the mothers was not statistically significant. An earlier study by Wayte et al^4^ showed a moderate relationship between the sleep problems of mothers and children (r=0.38, *p*=0.016), similar to our study (r=0.36, *p*>0.05) except their observed correlation was statistically significant.^4^ Their study sample consisted of 57 children with CP, so our smaller sample size may not have been sufficient to show this relationship as statistically significant. In support of the previous work, we have also demonstrated a relationship between depression in mothers and their sleep quality as well as significantly correlations between maternal depression and the children’s sleep quality.

One of the weaknesses of our study was not to evaluate the sleep quality of children and mothers using the gold standard technique, polysomnography. However, both scales for assessing the sleep quality of mothers and children with CP have previously been validated against this gold standard. Other important points are the cross-sectional design of our study and having non-match control group of children with spastic CP, limiting the possibility of building a direct link between the BoNT-A injection and the improvement in the children and the mothers’ sleep quality and depression. One strength of our study is to have the multiple time point assessments of participants, which allowed assessment of the changing effect of BoNT-A over time.

In conclusion, our study suggests that the BoNT-A injection for spasticity in children with CP may have the potential to improve sleep quality in children with CP and their primary caregiver, the mother, as well as to reduce depression in the mother. Further prospective randomized control studies with larger sizes are warranted to test these results.
